# Identification of CCT3 as a prognostic factor and correlates with cell survival and invasion of head and neck squamous cell carcinoma

**DOI:** 10.1042/BSR20211137

**Published:** 2021-10-19

**Authors:** Yan Wang, Peicheng Liu, Ziwei Zhang, Jiulong Wang, Zhigang Cheng, Chengchao Fan

**Affiliations:** Department of Oral and Maxillofacial Surgery, The Central Hospital of Wuhan, Tongji Medical College, Huazhong University of Science and Technology, Wuhan, China

**Keywords:** CCT3, cell survival, HNSCC, metastasis, prognostic factor

## Abstract

**Background:** Recurrent locally advanced or metastatic head and neck squamous cell carcinoma (HNSCC) is associated with dismal prognosis because of its highly invasive behavior and resistance to conventional intensive chemotherapy. The identification of effective markers for early diagnosis and prognosis is important for reducing mortality and ensuring that therapy for HNSCC is effective. Chaperonin-containing TCP-1 3 (CCT3) folds cancer-related proteins to control carcinogenesis. The prognostic value and growth association of CCT3 and HNSCC remain unknown.

**Methods:** The GEO, Oncomine and UALCAN databases were used to examine CCT3 expression in HNSCC. A few clinical HNSCC samples with normal tissues were used to detect CCT3 expression by using immunohistochemistry method. The TCGA-HNSC dataset was used to evaluate the association between expression of CCT3 and prognosis. The molecular mechanism was investigated with gene set enrichment analysis (GSEA). CCK-8 and wound healing assays were used to detect cell growth and invasion of HNSCC, respectively.

**Results:** CCT3 expression was significantly up-regulated in HNSCC in both mRNA and protein levels. In addition, up-regulated CCT3 expression was associated with various clinicopathological parameters. High expression of CCT3 was significantly correlated with inferior survival of HNSCC patients. Knockdown of CCT3 significantly inhibited cell growth and invasion of HNSCC cell lines. GSEA analysis indicated that CCT3 was closely correlated with tumor-related signaling pathways and HNSCC cell survival.

**Conclusion:** Our findings suggest that CCT3 is a biomarker of poor prognosis and related to the process of HNSCC.

## Introduction

Head and neck cancer (HNC) is a common malignant worldwide and causes more than 600,000 new cases every year [1]. Head and neck squamous cell carcinoma (HNSCC) accounts for the major pathological type of HNC [2]. Although rapid advances in methods of diagnosis and treatment, the prognosis of patients with HNSCC remains not optimistic [3]. Therefore, it is urgent to identify prognostic biomarkers for precise molecular diagnosis and potential targeted drug treatment in the future.

Chaperonins are kinds of proteins that help to fold stress-denatured polypeptide chains [4]. Two groups of chaperonins are defined: group I includes Heat shock protein 60 (HSP60) or GroEL in bacteria, and group II is Chaperonin-containing TCP-1 (CCT). CCT consists of eight distinct subunits, named CCT1-CCT8 [5,6]. CCT folds cancer-related proteins to control carcinogenesis, such as Signal transducers and activators of transcription 3 (STAT3), kirsten rat sarcoma viral oncogene (KRAS), and p53 [7–9].

CCT3 is widely studied in various cancers [10–16]. The expression of CCT3 is increased both at mRNA and protein level in hepatocellular carcinoma (HCC) tissues than those in non-HCC tissues, and CCT3 involves in carcinogenesis and development of HCC and has prognostic indication in HCC [10,11]. Similar results are found in gastric cancer [12]. In addition, knockdown of CCT3 decreased the cell viability of gastric cancer cells and accounted for inhibited expression of cell division cycle 42 (cdc42), mitogen-activated protein kinase 7, cyclin D3 and up-regulated of cyclin-dependent kinase 2 and 6 [12]. Silencing CCT3 inhibited the proliferation, stopped cell cycle and induced apoptosis in papillary thyroid carcinoma cells [13]. In breast cancer, Xu et al. have reported that CCT3 knockdown significantly inhibited activity of NF-κB and decreased the proliferation and metastasis ability of breast cancer cells [14,15]. Recently, a study performed a bioinformatic analysis focusing on the relationship between CCTs and HNSCC which indicated that CCT3 might be a potential biomarker for prognosis of HNSCC patients [16]. Hence, we further explored the association between CCT3 and HNSCC.

In the present study, we have evaluated the relationship between CCT3 and HNSCC using analysis of comprehensive bioinformatics methods and basic experiments. CCT3 was overexpressed in tumor tissues of HNSCC than corresponding normal tissue. Increased CCT3 was significantly associated with process of HNSCC and suggested a poor prognosis in patients. Importantly, knockdown of CCT3 inhibited cell growth and cell migration of HNSCC cells.

## Methods and materials

### Cell cultures

The human tongue squamous cell carcinoma cell lines SCC25 and CAL27 were kindly provided by Prof. Juan Lv (Taihe Hospital, Shiyan) [17]. SCC25 and CAL27 were grown in complete DMEM (Hyclone, U.S.A.) containing 10% fetal bovine serum (FBS, Lonza, U.S.A.) with 100 units/ml streptomycin and penicillin (Life Technologies). All cells were cultured in 37°C with 5% CO2. All cells were harvested using 0.25% trypsin-EDTA (Life Technologies) when growing to 80% density.

### Cell transfection

Cell lines SCC25 and CAL27 were transfected with the special small interfering RNA (siRNA, CCT3, 5′-UGAAAGUAAAGUAUUCAUCUCGAUGAAUACUUUACUUUCAUC-3′;) or a non-specific control using Lipofectamine 2000 Transfection Reagent (Sigma-Aldrich) according to the manufacturer’s instructions.

### Western blotting

The cell lysates of SCC25 and CAL27 cell lines were collected at 80–90% density. Twenty micrograms of total protein were fractionated with 10% SDS-page gel and transferred to 0.2 μm PVDF membrane (Biorad) at 100 V for 80 min. After being blocked with PBS containing 0.1% Tween 20 and 5% low-fat milk for 1 h at room temperature, the PVDF membrane was incubated with primary antibody overnight at 4°C. The next day, after being washed three times with TBST, the membrane was cultured with secondary antibody for 1 h at room temperature and subjected into ECL developing system. We used the rabbit polyclonal CCT3 antibody (cat no. ab244288, 1:1000, Abcam, U.K.) and mouse monoclonal anti-GAPDH (cat no. ab8245, 1:10000, Abcam, U.K.).

### Cell viability

After transfection with siRNA, 1 × 10^4^ SCC25 and CAL27 cell lines were collected and seeded in 96-well plates. Then cells were incubated with complete medium for 24 or 48 h. Ten micrograms of CCK-8 reagent (MedChemExpress) were added into each well. Cells were incubated in 37°C for 30 min and subjected to read OD value within 450 nm absorbancy.

### Wound healing

SCC25 and CAL27 were seeded and transfected in 12-well plate at 1 ml/well medium. After grown 80–90% density, cells were washed by PBS and were scratched slightly with a 200 μl pipette tip. The wound area was considered as orientation and photographed at 0 h. After washed with PBS for thre times, cells were incubated with DMEM without FBS for 48 h. Then, the wounds were photographed and quantified. The wound sizes were determined by measuring the width of wound at 48 h and subtracted by the wound width at 0 h [18].

### Tissue sample and immunohistochemistry (IHC)

Totally, 47 patients with HNSCC (47 tumor samples and 15 normal controls) were included in this study. The involved tumor patients were come from the Central Hospital of Wuhan and definitely diagnosed in department of pathology since 2017. The expression and distribution of CCT3 in HNSCC was detected by IHC. Briefly, the paraffin-embedded sections were deparaffinized, restored and quenched. After blocking with goat serum for 1 h, sections were incubated with primary antibodies against CCT3 (cat no. ab244288, 1:1000, Abcam, U.K.) overnight at 4°C. Then, the sections were washed with PBS and incubated with secondary antibody (MaxVision™ Kits, MXB, China) with horseradish peroxidase-conjugated polymer for 15 min. Subsequently, tissue section was stained with a DAB for 1 min. Finally, sections were counterstained with Harris hematoxylin for 20 s.

### Analyzing the expression of CCT3 in HNSCC

The Sangerbox database (http://sangerbox.com/) provides dependent tools allowing users to perform customized bioinformatics analysis. The Sangerbox was used to analyze the mRNA expression of CCT3 in pan-cancer of TCGA database and GEO database (GSE13398, GSE29330 and GSE136037). GSE13398 has 16 paired mRNA-seq data which calculated with a GPL7540 platform. GSE29330 includes 18 unpaired samples analyzed with the GPL570 platform. GSE136037 contains 72 HNSCC samples with clinical TNM information. These datasets were normalized through RMA method. UALCAN provides systematical analysis of TCGA gene expression data (http://ualcan.path.uab.edu), which allows clients performing in-depth and personalized analyzing. The expression of CCT3 was determined in the ‘TCGA-HNSC’ dataset and with the ‘Expression Analysis’ module. Furthermore, the mRNA expression of CCT3 between HNSCC and normal samples based on diverse clinicopathological parameters was also analyzed using the UALCAN database. ONCOMINE (www.oncomine.org) is currently the largest oncogene chip database which contains 715 gene expression data sets and contains independent integrated data mining function. Detailed expression of CCT3 in different primary sites of HNSCC was investigated using ONCOMINE.

### The prognosis evaluation of CCT3 in HNSCC

The Kaplan–Meier plotter (www.kmplot.com) is capable of evaluating the prognostic values of 54,000 genes in 21 types of cancer. The correlation of mRNA expression of CCT3 and prognosis of HNSCC patients was analyzed using the Kaplan–Meier plotter as well as the prognostic values based on diverse clinicopathological characteristics. To analyze the survival events, total cases were automatically divided into two groups based on getting an available outcome with computer. The hazard ratio (HR) with 95% confidence intervals and log-rank *P*-value were obtained to evaluate the significance of prognosis in HNSCC patients.

### The interaction network and co-expression analysis of CCT3

To construct the protein–protein interaction (PPI) network of CCT3, GeneMANIA (http://www.genemania.org) and STRING (https://string-db.org/) were used. GeneMANIA provides predication of protein interaction and develops an interactive functional-association network which contains a list of genes with similar functions. STRING provides PPI network analysis including both certified and predicated links. CCT3 (protein name) and Homo sapiens (organism) were chosen. GEPIA database is a comprehensive web-portal which allows users to perform a single or multiple gene analysis based on TCGA datasets. We explore the associations of CCT3 and other key candidate genes using the ‘Correlation Analysis’ module.

### Gene Set Enrichment Analysis (GSEA) of CCT3

GSEA (http://www.broad.mit.edu/gsea)was performed to annotate the Hallmark and KEGG effector gene sets associated with the mRNA expression of CCT3 in the TCGA-HNSC dataset. The FDR<0.25 and *P*<0.05 was considered as significant.

### Identification of essential role of CCT3 for cell survival

Project Achilles comprehensively scores and identifies total 18000 genes which whether essential for cell survival in numbers of characterized cancer cell lines. The original data of CERES score for HNSCC cell lines were obtained from Depmap portal (https://depmap.org/portal). The CERES score approaches to 0 means when the gene was removing with CRISPR-Cas9 system, the cell growth has not been inhibited significantly. When the score less than −1, it means that the gene is essential for cell survival in this cancer cell line.

### Statistical analysis

The hazard ratio (HR) with logrank *P* value was used to evaluate the significance of survival. Spearman’s correction was used to assess the association of gene expression and the strength of the correlation. Student’s *t*-test was used to evaluate the significance between two groups and triple repetitive experiments. The chi-square test was used to detect the significance of expression of CCT3 and clinicopathologic features. Results were considered as statistically significance at *P<0.05, **P<0.01 and ***P<0.001.

## Results

### Expression of CCT3 in HNSCC

**Figure 1:**
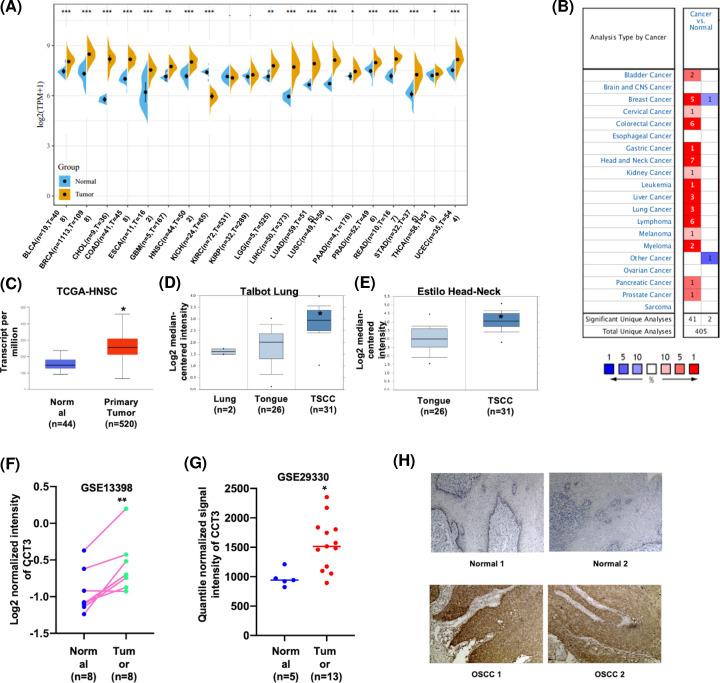
The expression of CCT3 was increased in HNSCC samples (**A**) CCT3 expression profiles in various types of cancer in the Sangerbox database. (**B**) The mRNA transcriptional levels of CCT3 in different types of cancers in the ONCOMINE database. The numbers in the box suggested statistically significant (*P* < 0.0001) of overexpressed (red) or down-expressed (blue) of CCT3 mRNA in cancer tissues compared with normal tissues. (**C**) Expression of CCT3 in HNSCC tissues compared with that in normal tissues from the UALCAN database. Separate box plots comparing CCT3 expression in normal and HNSCC tissues from the analysis of Talbot Lung (**D**), Estilo Head-Neck (**E**) and Pyeon Muti-cancer. The expression of CCT3 in GSE13398 (**F**) and GSE29330 (**G**) datasets. (**H**) IHC staining of CCT3 in normal oral and OSCC tissues was analyzed. Normal1 and Normal2: adjacent normal tongue tissue; OSCC1 and OSCC2: OSCC tissue; *, *P* < 0.05; **, *P* < 0.01; ***, *P* < 0.001; CCT3, Chaperonin-containing TCP-1 3; HNSCC, head and neck squamous-cell carcinoma; OSCC, oral cavity squamous-cell carcinoma; TSCC, tongue squamous-cell carcinoma

To determine the expression of CCT3 in various cancers, TCGA and Oncomine databases were explored. As shown in Figure 1A, the mRNA level of CCT3 was expressed higher in most cancer tissues including: bladder urothelial carcinoma (BLCA), breast invasive carcinoma (BRCA), cholangiocarcinoma (CHOL), colon adenocarcinoma (COAD), esophageal carcinoma (ESCA), glioblastoma multiforme (GBM), HNSCC, kidney renal clear carcinoma (KIRC), kidney renal papillary carcinoma (KIRP), liver hepatocellular carcinoma (LIHC), lung adenocarcinoma (LUAD), brain lower grade glioma (LGG), lung squamous cell carcinoma (LUSC), pancreatic adenocarcinoma (PAAD), prostate adenocarcinoma (PRAD), Rectum Carcinoma (READ), stomach adenocarcinoma (STAD), thyroid carcinoma (THCA) and uterine corpus endometrial carcinoma (UCEC) than in corresponding normal tissues. In kidney chromophobe (KICH), the expression of CCT3 was higher in normal tissues than in tumor tissues. Then, the expression of CCT3 in tumor tissues was compared with which in normal samples by using ONCOMINE databases (Figure 1B). The mRNA profiles of CCT3 were increased in different types of cancers versus the corresponding normal tissue. Because CCT3 was significantly up-regulated in HNSCC in seven datasets, the association between CCT3 and HNSCC was subjected into subsequent exploration. The CCT3 expression of TCGA-HNSC in normal and tumor tissue was presented in Figure 1C. As shown in Figure 1D,E, the expression of CCT3 in tongue squamous cell carcinoma (TSCC, Talbot lung and Estilo Head-Neck, Oncomine) were all significantly up-regulated than in normal tongue tissues. To further confirm these results, one paired (GSE13398) and one non-paired (GSE29330) GEO datasets were explored. As shown in Figure 1F,G, CCT3 expression was significantly increased in tumor tissues than in normal oral samples. We further investigated the protein expression of CCT3 in HNSCC using IHC assay. Total 47 tumor samples and 15 normal oral tissues were analyzed. The expression of CCT3 was mainly accumulated in cytoplasm. The protein expression of CCT3 was significantly higher in OSCC than in normal epithelial tissues (Figure 1H). These results suggested that CCT3 was highly expressed in HNSCC than in associated normal tissues.

### The prognostic analysis of CCT3 in HNSCC

**Figure 2:**
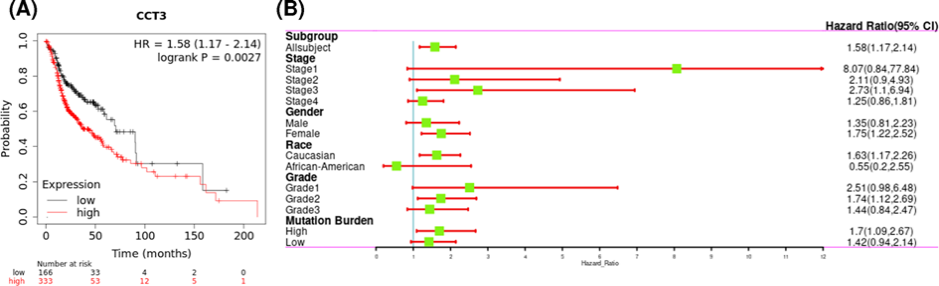
CCT3 expression is positively associated with poor prognosis of patients with HNSCC (**A**) OS within TCGA-HNSC were analyzed based on mRNA expression of CCT3 using Kaplan–Meier plotter. HR with 95%CI and *P* value were used to estimate the significance of CCT3 on prognosis of patients with OSCC. If HR > 1, it means that high expression of CCT3 was associated with a poor prognosis. (**B**) Forest plots showing the association between CCT3 expression and clinicopathological features in HNSCC patients; CCT3, Chaperonin-containing TCP-1 3; CI, confidence intervals; HR, hazard ratio

To explore whether CCT3 is associated with the prognosis of HNSCC patients, the OS of TCGA-HNSC dataset was investigated by using Kaplan–Meier plotter. As shown in Figure 2A, high expression of CCT3 indicated poor prognosis in HNSCC patients [OS, HR = 1.58, (1.17–2.14), *P*=0.0027]. To understand the affection of CCT3 on prognosis of HNSCC, the association between expression profiles of CCT3 based on different clinical features and prognostic value of HNSCC patients was investigated. As shown in Figure 2B, high expression of CCT3 suggested worse prognosis in subgroup analysis of stage 1, stage 3, male, Caucasian, grade 1, grade 2 and high mutation burden (*P*<0.05). These results suggested that clinical stage, tumor grade, gender, race and mutation burden have some influences on the effect of CCT3 in HNSCC.

### Association of CCT3 expression and clinicopathological characteristics

**Figure 3:**
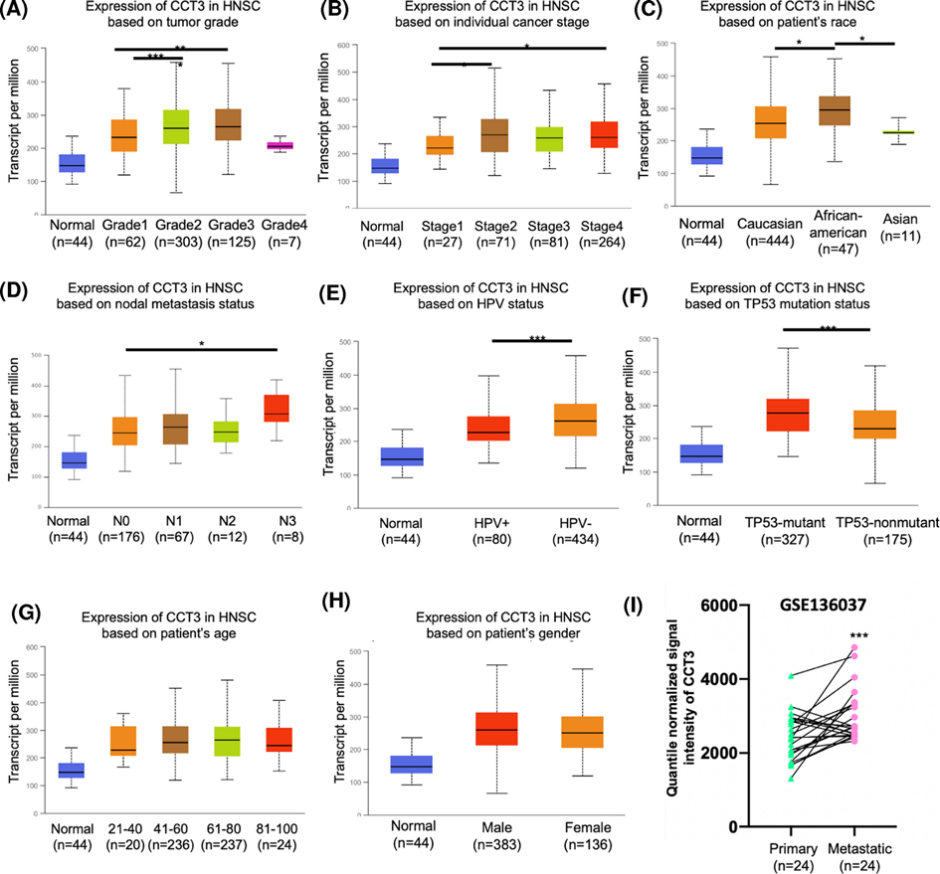
Expression of CCT3 plays important role in process of HNSCC CCT3 expression was analyzed by (**A**) tumor grade (from grade 1 to grade 4), (**B**) clinical stages (from stage 1 to stage 4), (**C**) patient’s race, (**D**) nodal metastasis statuses (from N0 to N3), (**E**) HPV status, (**F**) TP53 mutation status, (**G**) patient’s age and (**H**) gender. (**I**) The expression of CCT3 in metastatic and primary HNSCC tissues was detected in GSE136037 dataset. *, *P* < 0.05; ***, *P* < 0.001; CCT3, Chaperonin-containing TCP-1 3; HNSCC, head and neck squamous-cell carcinoma; HPV, human papillomavirus

Since CCT3 expression was significantly increased in HNSCC tissues, we further explore the expression profiles of CCT3 based on clinicopathological parameters. The TCGA-HNSC datasets was analyzed by using the UALCAN database. Regarding the tumor grade, significant up-regulation of CCT3 expression was found in grade 2 and grade 3 patients than in grade 1 patients (Figure 3A). In addition, the expression of CCT3 was up-regulated in stage 2 and stage 4 of HNSCC than in stage 1 (Figure 3B). The CCT3 expression was significantly increased in African-american than in Caucasian and Asian (Figure 3C). Regarding the nodal metastasis status, the mRNA expression of CCT3 was significantly up-regulated in N3 patients with HNSCC than in N0 (Figure 3D). Compared with HPV+ patients, CCT3 was highly expressed in HPV- patients (Figure 3E). In addition, the CCT3 expression was upregulated in TP53-mutat patients than in TP53-normal patients (Figure 3F). Expression of CCT3 was not associated with patient’s age (Figure 3G) and gender (Figure 3H). Because CCT3 was associated with nodal metastasis, the expression of CCT3 was investigated in GSE136037 dataset. The results showed that up-regulated CCT3 was found in metastatic tumor tissues compared with primary tumor tissues (Figure 3I). These results suggested that CCT3 may be associated with more serious process of illness.

### CCT3 expression is associated with cell survival and invasion of HNSCC

**Figure 4:**
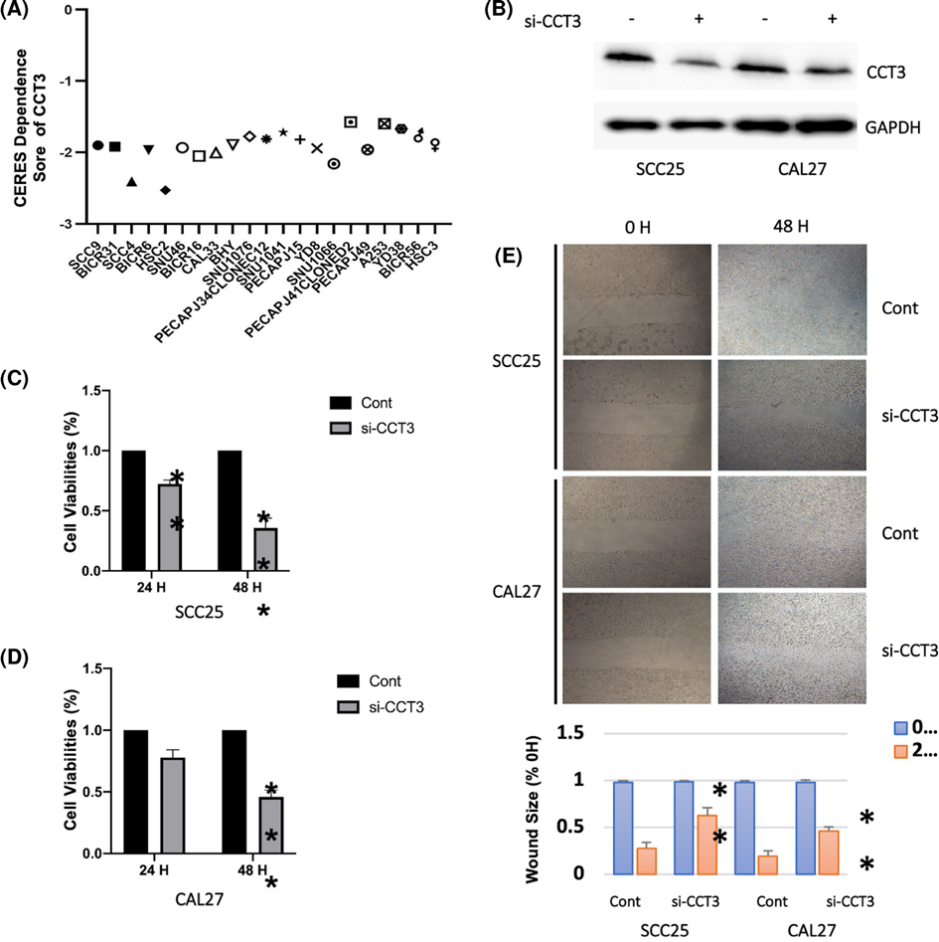
CCT3 is critical for cell survival and invasion of HNSCC cells (**A**) CERES score was obtained from Depmap datasource and used to access the significance of CCT3 for cell survival of HNSCC. CERES score approach to -1 means the gene is important for cell survival, while score approach to 0 means the gene is not an essential gene. (**B**) SCC25 and CAL27 cells were transfected with control siRNA or siRNA-CCT3 for 48 h. Cells were harvested and subjected to Western blot assay to detect the expression of CCT3. A CCK-8 assay was performed to detect cell viability of SCC25 (**C**) and CAL27 (**D**) cells after being transfected with siRNA. (**E**) A wound healing assay was performed to determine the cell invasion of SCC25 and CAL27 cells after being transfected with siRNA. The cells were photographed at 0 or 48 h; *, *P* < 0.05; **, *P* < 0.01; ***, *P* < 0.001; CCT3, Chaperonin-containing TCP-1 3.

Since CCT3 was associated with the progress of HNSCC, we investigated the CERES dependence scores of HNSCC cell lines to determine the importance of CCT3 for survival of tumor cells. As shown in Figure 4A, when removing the expression of CCT3 genes with CRISPR-Cas9 system, total 21 HNSCC cell lines got the scores less than -1, which suggested that CCT3 plays a critical role for HNSCC cell survival. To further confirm these results, the sham siRNA or siRNA-CCT3 was transfected to SCC25 or CAL27 cell lines. As shown in Figure 4B, CCT3 was knockdown in both SCC25 and CAL27 cell lines. After transfected with siRNA, cells were subjected to a CCK-8 assay to detect cell viabilities at 24 or 48 h. As shown in Figure 4C, knockdown of CCT3 significantly inhibited cell viability of SCC25 cells at 24 h checkpoint. In addition, down-regulated CCT3 decreased cell viabilities of SCC25 and CAL27 cells after 48 h transfection (Figure 4C,D). Then, a wound healing assay was performed to detect the cell invasion affected by CCT3. As shown in Figure 4E, after being transfected with siRNA-CCT3, the wound sizes were significantly decreased in both SCC25 and CAL27 cell lines. These results suggested that CCT3 was important for cell growth and invasion of HNSCC cells.

### The gene set enrichment analysis of CCT3 in HNSCC

**Figure 5:**
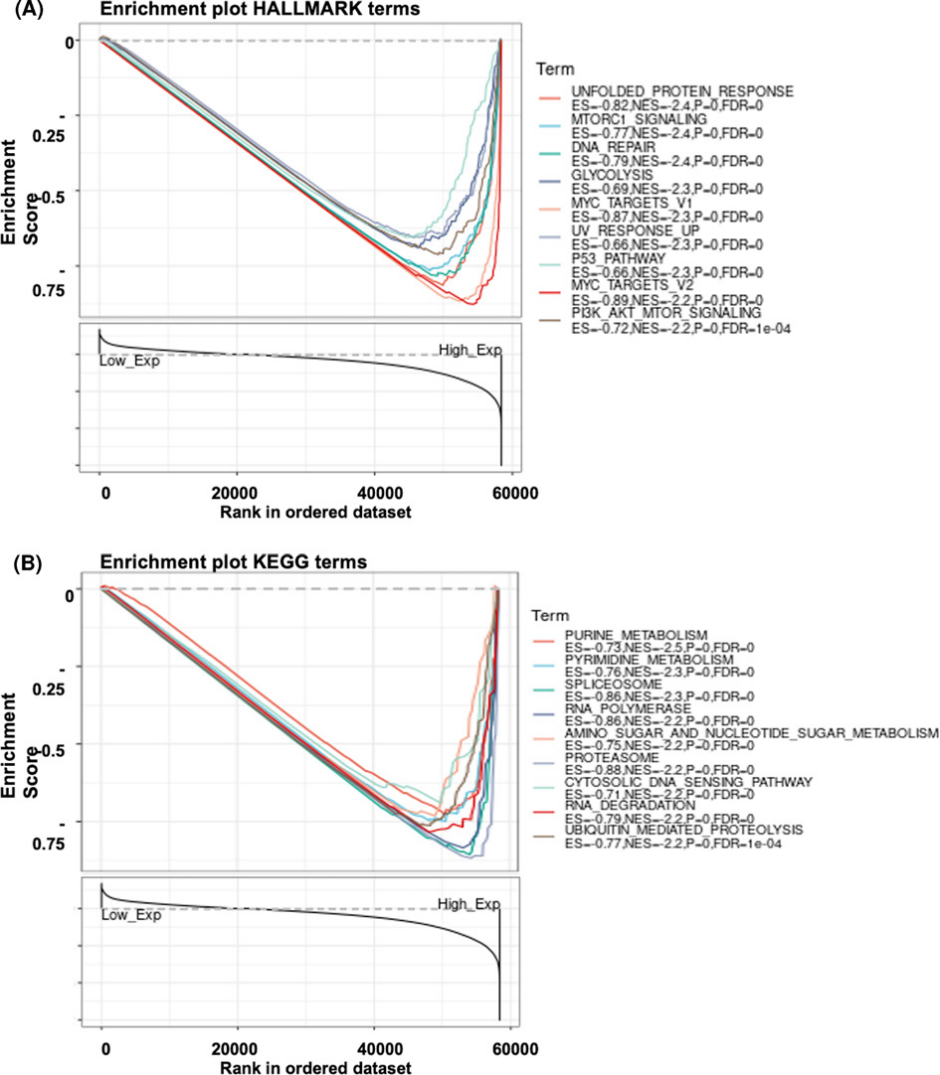
The Gene Set Enrichment Analysis of CCT3 in HNSCC GSEA analysis was performed with Hallmark (**A**) and KEGG (**B**) pathways based on CCT3 expression. The most top 9 affected signal pathways were included in the graphics; CCT3, Chaperonin-containing TCP-1 3; GSEA, Gene Set Enrichment Analysis; HNSCC, head and neck squamous cell carcinoma

To evaluate the possible mechanism of CCT3 on HNSCC, GSEA with the annotation of Hallmark and KEGG gene sets was performed. Total 43 and 131 important pathways were significantly affected by high expression of CCT3 in Hallmark and KEGG analysis, respectively (Supplementary Tables S1 and 2, NOM *P*-val < 0.05, FDR *q*-val< 0.25). The top 9 critical pathways of Hallmark (Figure 5A, UNFOLDED_PROTEIN_RESPONSE; MTORC1_SIGNALING; DNA_REPAIR; GLYCOLYSIS; MYC_TARGETS_V1; UV_RESPONSE_UP; PATHWAY; MYC_TARGETS_V2; PI3K_AKT_MTOR_SIGNALING) and KEGG (Figure 5B, PURINE_METABOLISM; SPLICEOSOME, PYRIMIDINE_METABOLISM; AMINO_SUGAR_AND_NUCLEOTIDE_SUGAR_METABOLISM; RNA_POLYMERASE; PROTEASOME; RNA_DEGRADATION; CYTOSOLIC_DNA_SENSING_PATHWAY; UBIQUITIN_MEDIATED_PROTEOLYSIS) were shown.

### Identification of key candidate genes from the CCT3 interaction network

**Figure 6:**
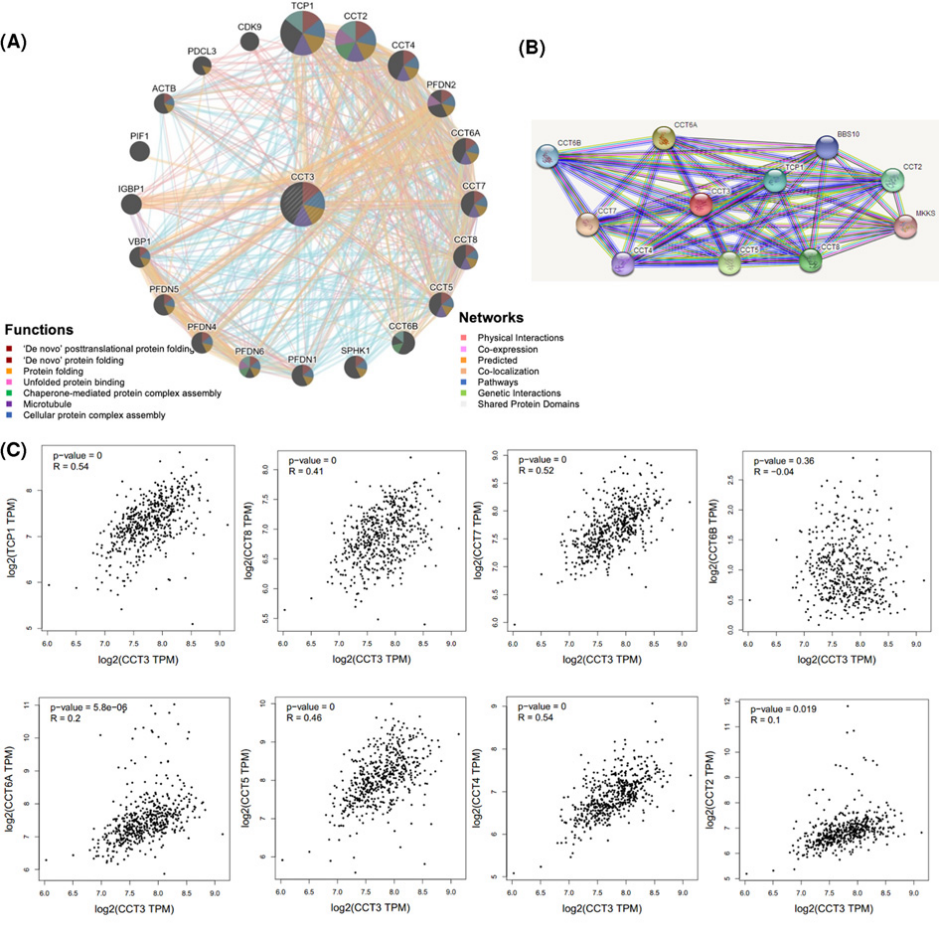
Identification of key candidate genes from the CCT3 interaction network (**A**) The gene–gene interaction network of CCT3 was obtained from the GeneMANIA database. Each node represents a gene. Total 20 most associated neighboring genes are shown. Different biological functions are presented by corresponding colors in the nodes. (**B**) The PPI network of CCT3 was constructed using the STRING database. (**C**) Scatterplots of correlations between CCT3 with TCP1, CCT8, CCT7, CCT6B, CCT6A, CCT5, CCT4 and CCT2 in HNSCC; CCT, Chaperonin-containing TCP-1

To explore mechanisms of CCT3 in HNSCC and analyzed the function of these genes, a gene–gene interaction network for CCT3 was constructed using the GeneMANIA database. Total 20 nodes surrounding CCT3 represented genes that were significantly associated with CCT3 (Figure 6A). The tightest corrected 5 genes were TCP1, CCT2, CCT4, PFDN2 and CCT6A. Additional functional analysis suggested that the proteins encoded by these genes were dramatically related with the following terms: ‘De novo’ post-translational protein folding, ‘De novo’ protein folding, Protein folding, Unfolded protein binding, Chaperone-mediated protein complex assembly, Microtubule and Cellular protein complex assembly. Then we constructed a PPI network using the STRING database to further explore the function of CCT3. A total of 10 CCT3-interacting proteins were included in the PPI network complex by filtering (Figure 6B). Importantly, eight common hub genes were identified from the GeneMANIA and STRING databases: TCP1, CCT8, CCT7, CCT6B, CCT6A, CCT5, CCT4 and CCT2. Co-expression analysis between CCT3 and these five interacting proteins were performed with the GEPIA database. As shown in Figure 6C, the expression of CCT3 was strongly correlated with that these genes except for CCT6B in HNSCC.

## Discussion

In the present study, we conducted a comprehensive bioinformatics analysis and clinical sample assay to indicate that the expression of CCT3 was higher in HNSCC tissues than in corresponding normal tissues both in mRNA and protein levels. Overexpressed CCT3 in HNSCC tissues suggested poorer prognosis than patients with low-expressed CCT3. Importantly, knockdown of CCT3 leaded to inhibition of growth and invasion in HNSCC cell lines. Above all, CCT3 plays an important role in the progress of HNSCC.

The abnormal expression of CCT3 has been proved to influent the migration of tumor cells and the prognosis of cancer patients in previous researches. High expression of CCT3 was found in some cancers, such as hepatocellular carcinoma, multiple myeloma, colorectal cancer, liver cancer and gastric cancer [12,19–22], and increased expression of CCT3 was not only indicated a poor prognosis in patients with hepatocellular carcinoma but also correlated with lymph-node metastasis of gastric cancer [23,24]. Similar with these studies, our analysis suggested that overexpressed CCT3 was found in HNSCC tissues, and high level CCT3 indicated an inferior prognosis of patients with HNSCC. The mRNA expression of CCT3 was significantly up-regulated in patients with more nodal metastasis status.

CCT3 takes part in the folding process of nearly 7% cellular proteins, such as cyclin E, cytoskeletal proteins (tubulins, actins) and Von Hippel-Lindau (VHL), which determines the central role of CCT in the growth of malignant cells [6,25,26]. Previous studies have reported the inhibition of CCT3 expression can suppress the proliferation of various cancer cells, such as papillary thyroid carcinoma, gastric carcinoma, breast cancer and hepatocellular carcinoma [12–14,27]. In our study, the results of CERES dependence scores suggested that CCT3 was important for cell survival of HNSCC. In addition, knockdown of CCT3 with a special siRNA in HNSCC cell lines leaded to the growth suppression of cancer cells, which is consistent with studies in other cancers [12–14,27]. Importantly, higher expression of CCT3 was found in nodal metastasis patients. In addition, the expression of CCT3 in metastatic lesions was higher than in primary tumor tissues. These results suggested that CCT3 may be associated with metastatic capacity of HNSCC. Consistently, the wound healing assay indicated the inhibition of cell invasion when knockdown of CCT3 expression. Above all, CCT3 might be a potential drug target in HNSCC.

In clinical subgroup analysis, expression of CCT3 was associated with higher cancer stages and tumor grades. Regarding mechanisms, CCT3 affects the progression of HCC by activating signal transducer and activator of transcription 3 (STAT3) [10,28]. STAT3 is the major factor in JAK-STAT3 pathway signaling, which plays an important role in many aspects of tumorigenesis [29]. The activation of STAT dimers in nucleus can be affected by mitogen-activated protein kinase (MAPK), AKT/mammalian target of rapamycin (mTOR) and JAK [30], and a recent study disclosed that mTORC, which is multi-protein signaling complex of mTOR, assembly and signaling can be affect by eukaryotic chaperonin CCT [31]. Qian et al. has suggested that CCT3 expression was associated with JAK-STAT3 rather than mTOR pathway by KEGG and GSEA analysis [21]. However, in the present study, our results through GSEA analysis indicated that high expression of CCT3 was associated mTOR pathway (MTORC1/PI3K_AKT_mTOR). In addition, CCT3 gene was positively associated with high expression of MYC in breast cancer, which was also consistent with our findings in Hallmark analysis. [32] For other carcinogenesis associated pathways, overexpression of CCT3 was associated with unfolded protein response, DNA repair and p53 pathway, which may contribute to the progress of HNSCC.

It should be acknowledged that there were some shortcomings and limitations in this study. First, although the mRNA and protein expression of CCT3 were analyzed in HNSCC by using multiple public resources and a few clinical samples, respectively. It’s still lacking enough clinical data to analysis the association between CCT3 and the process of HNSCC. Second, the correlation between CCT3 expression and prognosis of HNSCC patients was not strong. High expression of CCT3 indicated only poorer OS but not other prognostic indicator. In addition, lacking more large public datasets, especially for some little cohorts, is a significant shortcoming. Third, more molecular experiments should be performed to uncover how the expression of CCT3 regulates growth and invasion of tumor cells to affect the progress of HNSCC *in vivo* and *in vitro*.

In summary, expression of CCT3 was up-regulated in HNSCC and significantly correlated with the clinicopathologic stages. Overexpression of CCT3 suggested poor prognosis of HNSCC patients. CCT3 was related with changes of carcinogenesis pathways, which were involved in the HNSCC growth and development. Moreover, knockdown of CCT3 significantly inhibited cell growth and invasion of HNSCC cell lines. Thus, CCT3 could be a prognostic marker and potential therapeutic target in HNSCC.

## Supplementary Material

Supplementary Tables S1-S2

## Data Availability

Sangerbox database (http://sangerbox.com/) UALCAN (http://ualcan.path.uab.edu) ONCOMINE (www.oncomine.org) HPA (https://www.proteinatlas.org/) Kaplan-Meier plotter (www.kmplot.com) GeneMANIA (http://www.genemania.org) STRING (https://string db.org/) GSEA (http://www.broad.mit.edu/gsea) Depmap portal (https://depmap.org/portal)

## Abbreviations

BLCA: bladder urothelial carcinoma
BRCA: breast invasive carcinoma
CCT3: Chaperonin-containing TCP-1 3
CHOL: cholangiocarcinoma
COAD: colon adenocarcinoma
ESCA: esophageal carcinoma
GBM: glioblastoma multiforme
GSEA: gene set enrichment analysis
HNSCC: head and neck squamous cell carcinoma
HSP60: Heat shock protein 60
IHC: Immunohistochemistry
MAPK: mitogen-activated protein kinase
mTOR: mammalian target of rapamycin
PPI: protein–protein interaction
KICH: kidney chromophobe
KIRC: kidney renal clear carcinoma
KIRP: Kidney Renal Papillary Carcinoma
LGG: brain lower grade glioma
LIHC: liver hepatocellular carcinoma
LUAD: Lung Adenocarcinoma
LUSC: lung squamous cell carcinoma
PAAD: pancreatic adenocarcinoma
PRAD: prostate adenocarcinoma
READ: PRAD
STAD: stomach adenocarcinoma
STAT3: signal transducer and activator of transcription 3
THCA: thyroid carcinoma
UCEC: uterine corpus endometrial carcinoma
VHL: Von Hippel-Lindau
