# Supplementary material for: Identification of CCT3 as a prognostic factor and correlates with cell survival and invasion of head and neck squamous cell carcinoma
Source: Biosci Rep. 2021 Oct 19;41(10):BSR20211137. doi: 10.1042/BSR20211137 (PMC8529339; doi:10.1042/BSR20211137)
Supplement: Supplementary Tables S1-S2 [file BSR-2021-1137_supp.pdf]

Supplemental Table 1: High expression of CCT3 affected signal pathways by GSEA analyzing (Hallmark)

NAME	SIZE	ES	NES	NOM p-val	FDR q-val
HALLMARK	110	-0.81949	-2.40671	0	0
HALLMARK	198	-0.76839	-2.37045	0	0
HALLMARK	148	-0.7873	-2.35289	0	0
HALLMARK	197	-0.69271	-2.3478	0	0
HALLMARK	197	-0.86934	-2.34562	0	0
HALLMARK	156	-0.66056	-2.31008	0	0
HALLMARK	197	-0.65778	-2.25089	0	0
HALLMARK	58	-0.88773	-2.23328	0	0
HALLMARK	104	-0.71886	-2.19514	0	1.11E-04
HALLMARK	160	-0.63716	-2.13637	0	3.23E-04
HALLMARK	-0.61791	-2.09386	0	9.58E-04	0.009
HALLMARK	-0.58962	-2.05958	0	0.001109	0.01
HALLMARK	-0.63115	-2.05716	0	0.001109	0.01
HALLMARK	-0.79279	-2.0601	0	0.001189	0.01
HALLMARK	-0.6112	-2.06415	0	0.001198	0.01
HALLMARK	-0.6279	-2.04627	0	0.001239	0.012
HALLMARK	-0.74367	-2.0669	0	0.001298	0.01
HALLMARK	-0.75289	-2.03107	0	0.001307	0.013
HALLMARK	-0.68759	-2.027	0.001901	0.001362	0.015
HALLMARK	-0.61445	-2.03346	0	0.00138	0.013
HALLMARK	-0.79799	-2.01453	0	0.001381	0.017
HALLMARK	-0.73132	-2.01472	0	0.001443	0.017
HALLMARK	-0.65178	-2.01945	0	0.001454	0.016
HALLMARK	-0.54693	-1.99292	0	0.001758	0.021
HALLMARK	-0.67676	-1.97962	0.001894	0.001822	0.023
HALLMARK	-0.64372	-1.95247	0	0.002489	0.028
HALLMARK	-0.53369	-1.94042	0	0.002754	0.032
HALLMARK	-0.69874	-1.92026	0.001942	0.003275	0.041
HALLMARK	-0.645	-1.88836	0.001873	0.004815	0.057
HALLMARK	-0.57118	-1.86491	0.007843	0.005751	0.074
HALLMARK	-0.53449	-1.86703	0	0.005765	0.071
HALLMARK	-0.58855	-1.83838	0.007678	0.007409	0.088
HALLMARK	-0.53941	-1.82654	0.005803	0.008124	0.097
HALLMARK	-0.51867	-1.80532	0.005859	0.009476	0.113
HALLMARK	-0.64618	-1.79425	0.022814	0.009922	0.125
HALLMARK	-0.58841	-1.79843	0.011834	0.009942	0.121
HALLMARK	-0.55862	-1.69679	0.031311	0.019898	0.211
HALLMARK	-0.71158	-1.68797	0.041905	0.021164	0.223

NOM p-val < 0.05 and FDR q-val < 0.25 were considered as significant

Abbreviations: GSEA, gene set enrichment analysis; CCT3, Chaperonin-containing TCP-1 3

Supplemental Table 2: High expression of CCT3 affected signal pathways by GSEA analyzing (KEGG)

NAME	SIZE	ES	NES	NOM p-val	FDR q-val
KEGG_PUR	154	-0.73146	-2.42507	0	0
KEGG_SPL	126	-0.86302	-2.30338	0	0
KEGG_WNT	150	-0.64845	-2.12539	0	8.51E-04
KEGG_FC_	93	-0.70262	-2.15193	0	8.72E-04
KEGG_EPI	68	-0.70929	-2.13676	0	9.01E-04
KEGG_GLY	72	-0.63627	-2.12571	0	9.08E-04
KEGG_SNA	38	-0.79504	-2.15462	0	9.51E-04
KEGG_CEL	124	-0.77011	-2.14323	0	9.70E-04
KEGG_PAT	56	-0.73452	-2.12079	0	9.81E-04
KEGG_AMY	53	-0.65803	-2.08018	0	0.001014
KEGG_PRO	45	-0.88383	-2.21339	0	0.00102
KEGG_RNA	29	-0.85995	-2.21671	0	0.001024
KEGG_RNA	56	-0.79322	-2.19518	0	0.001028
KEGG_MTO	51	-0.7052	-2.08894	0	0.001029
KEGG_CYS	34	-0.70983	-2.10545	0	0.001034
KEGG_PRO	85	-0.69828	-2.12191	0	0.001039
KEGG_OOC	111	-0.71305	-2.16787	0	0.001046
KEGG_NUC	44	-0.80591	-2.08161	0	0.001052
KEGG_NEU	125	-0.67545	-2.06746	0.001919	0.001056
KEGG_AMI	41	-0.84071	-2.09148	0	0.00107
KEGG_RIG	70	-0.64288	-2.0613	0	0.001078
KEGG_PAT	322	-0.62187	-2.10658	0	0.001086
KEGG_HUN	167	-0.66524	-2.06971	0	0.001093
KEGG_GLY	25	-0.8035	-2.05237	0	0.001107
KEGG_VIB	53	-0.71127	-2.05489	0	0.001109
KEGG_END	178	-0.66695	-2.0934	0	0.001115
KEGG_PRO	23	-0.82852	-2.04599	0.001901	0.001134
KEGG_BAS	35	-0.80976	-2.10663	0	0.001143
KEGG_UBI	130	-0.76804	-2.1714	0	0.001162
KEGG_VEG	76	-0.6445	-2.09433	0	0.001163
KEGG_LYS	121	-0.6966	-2.0468	0	0.001167
KEGG_REG	212	-0.63436	-2.09944	0	0.001171
KEGG_CYT	54	-0.70894	-2.18032	0	0.001173
KEGG_AMI	43	-0.75092	-2.22537	0	0.00128
KEGG_REN	66	-0.71693	-2.0394	0.001916	0.001301
KEGG_N_G	46	-0.7594	-2.04064	0	0.001337
KEGG_PYR	98	-0.75573	-2.27967	0	0.001348
KEGG_BAS	34	-0.80351	-2.02056	0	0.00175
KEGG_SMA	84	-0.67738	-2.01771	0	0.001838
KEGG_PHO	76	-0.66027	-1.98854	0	0.00224
KEGG_ERB	86	-0.65783	-1.99058	0	0.002244
KEGG_ALZ	152	-0.63184	-1.99355	0	0.002287
KEGG_PAN	69	-0.67612	-1.99083	0	0.002297
KEGG_CHR	73	-0.70753	-1.99538	0	0.002317
KEGG_GLI	65	-0.6523	-1.98011	0.003759	0.002406
KEGG_TOL	102	-0.61754	-1.97692	0.003766	0.00242

KEGG_P53	66	-0.6511	-1.97125	0.001927	0.00253
KEGG_T_C	107	-0.66293	-1.96829	0.003774	0.002661
KEGG_APO	87	-0.65272	-1.9629	0.003774	0.002763
KEGG_GAP	88	-0.60886	-1.95979	0.001869	0.002832
KEGG_HOM	26	-0.83246	-1.95725	0	0.002903
KEGG_INS	137	-0.60338	-1.95491	0	0.002932
KEGG_NOD	61	-0.66005	-1.94458	0.00381	0.003164
KEGG_GAL	26	-0.66311	-1.94518	0	0.003221
KEGG_B_C	75	-0.66489	-1.94531	0.001883	0.003281
KEGG_FC_	79	-0.61319	-1.93939	0	0.003298
KEGG_LYS	39	-0.72081	-1.94738	0	0.003305
KEGG_NON	54	-0.6622	-1.93199	0.003802	0.00358
KEGG_MAP	266	-0.56611	-1.92391	0	0.003858
KEGG_INO	54	-0.67306	-1.92262	0	0.003899
KEGG_THY	29	-0.69587	-1.91949	0.001905	0.003913
KEGG_GLY	16	-0.75837	-1.9205	0	0.003924
KEGG_COL	62	-0.66683	-1.92071	0.003817	0.003956
KEGG_NOT	47	-0.67618	-1.90623	0	0.004354
KEGG_ARG	53	-0.58251	-1.90698	0	0.004384
KEGG_OXI	115	-0.67651	-1.89779	0.001976	0.004419
KEGG_ADH	68	-0.68915	-1.89856	0.001961	0.004449
KEGG_PRO	89	-0.62706	-1.90163	0.007519	0.004469
KEGG_NON	12	-0.83952	-1.89938	0.003976	0.004476
KEGG_TIG	129	-0.58675	-1.90204	0	0.004517
KEGG_BLA	41	-0.63233	-1.89979	0	0.004526
KEGG_ONE	17	-0.75761	-1.88931	0	0.004964
KEGG_ACU	57	-0.6582	-1.88501	0.003854	0.005173
KEGG_GLY	43	-0.59111	-1.88097	0.003466	0.0053
KEGG_LON	70	-0.58599	-1.88313	0.00363	0.005309
KEGG_LON	68	-0.5635	-1.87873	0	0.005384
KEGG_SPH	36	-0.6646	-1.876	0	0.005419
KEGG_GLY	26	-0.66196	-1.86501	0.003788	0.005864
KEGG_FRU	33	-0.64785	-1.86219	0.001894	0.006085
KEGG_GNR	101	-0.5562	-1.85853	0	0.006093
KEGG_LEU	115	-0.57182	-1.85665	0.005556	0.006112
KEGG_POR	41	-0.63115	-1.85908	0.00349	0.006128
KEGG_RIB	87	-0.83177	-1.85723	0.005747	0.006142
KEGG_NAT	132	-0.57577	-1.85925	0.010969	0.00619
KEGG_MIS	23	-0.8104	-1.85951	0.001984	0.006254
KEGG_GLY	21	-0.70093	-1.85212	0	0.00633
KEGG_PEN	26	-0.66127	-1.84941	0	0.006574
KEGG_END	52	-0.65033	-1.84851	0.003802	0.006579
KEGG_SEL	25	-0.71045	-1.84327	0.001934	0.006724
KEGG_CIT	31	-0.73894	-1.83774	0.005803	0.007019
KEGG_GLU	49	-0.59949	-1.83246	0.007561	0.00711
KEGG_FOC	197	-0.61113	-1.83152	0.01341	0.007121
KEGG_AXO	128	-0.57708	-1.83352	0.009434	0.00715
KEGG_DNA	36	-0.81569	-1.83034	0.004049	0.007168
KEGG_REG	34	-0.59409	-1.83388	0.003731	0.007191

KEGG_ANT	81	-0.63798	-1.82611	0.015094	0.00751
KEGG_TGF	85	-0.59766	-1.82311	0.009398	0.007682
KEGG_VAS	43	-0.63641	-1.82358	0.003839	0.007694
KEGG_GLY	62	-0.57533	-1.81528	0.003738	0.008196
KEGG_PAR	114	-0.64042	-1.80154	0.014085	0.009181
KEGG_PER	78	-0.59375	-1.79988	0.007463	0.00924
KEGG_ADI	67	-0.57764	-1.79337	0.005607	0.009895
KEGG_PRI	35	-0.58679	-1.78981	0.008681	0.01015
KEGG_ALA	32	-0.60423	-1.77886	0.001934	0.011167
KEGG_DRU	51	-0.54159	-1.77145	0.003478	0.011734
KEGG_BAS	55	-0.57937	-1.76847	0.001898	0.012059
KEGG_SYS	133	-0.57066	-1.76173	0.019713	0.012688
KEGG_HED	56	-0.54939	-1.76184	0.001825	0.012797
KEGG_CHE	184	-0.55006	-1.75852	0.018692	0.013031
KEGG_JAK	155	-0.51932	-1.75641	0.01487	0.013194
KEGG_MEL	101	-0.52067	-1.7495	0.009042	0.014099
KEGG_OTH	16	-0.70982	-1.72051	0.003891	0.018788
KEGG_NIT	23	-0.60898	-1.72074	0.005515	0.0189
KEGG_STE	17	-0.69805	-1.72089	0.017308	0.019049
KEGG_VAL	11	-0.7587	-1.72128	0.001923	0.019075
KEGG_DOR	24	-0.62965	-1.71152	0.01107	0.019791
KEGG_GLY	22	-0.69702	-1.71289	0.02439	0.019846
KEGG_PYR	40	-0.5798	-1.67503	0.014815	0.025887
KEGG_PAN	16	-0.6416	-1.67548	0.014337	0.025943
KEGG_ETH	30	-0.54189	-1.67666	0.009294	0.026038
KEGG_MEL	71	-0.52666	-1.67176	0.016245	0.026382
KEGG_LEI	70	-0.59697	-1.66764	0.05	0.026873
KEGG_BIO	20	-0.64436	-1.65437	0.014493	0.029764
KEGG_GLY	15	-0.66313	-1.65178	0.022599	0.030224
KEGG_GLY	26	-0.54334	-1.645	0.016822	0.031332
KEGG_BET	22	-0.5835	-1.59523	0.03321	0.045202
KEGG_TER	15	-0.67257	-1.57704	0.056093	0.050415
KEGG_TYP	47	-0.49262	-1.55868	0.030741	0.056295
KEGG_FOL	10	-0.6653	-1.55597	0.022059	0.056906
KEGG_TYR	40	-0.50321	-1.54946	0.03777	0.058851
KEGG_GLY	14	-0.60897	-1.50851	0.054581	0.074042
KEGG_TRY	40	-0.4864	-1.50566	0.04529	0.074611
KEGG_CIR	13	-0.6625	-1.50166	0.085437	0.075694
KEGG_VIR	68	-0.52129	-1.4978	0.089866	0.076614
KEGG_RIB	15	-0.59439	-1.49005	0.049091	0.079373
KEGG_PRO	23	-0.52167	-1.48688	0.063521	0.080194
KEGG_VAS	113	-0.45526	-1.48183	0.07457	0.082012
KEGG_NIC	24	-0.50827	-1.46586	0.070076	0.088512
KEGG_GLY	15	-0.61055	-1.45639	0.097561	0.092009
KEGG_O_G	29	-0.51033	-1.44777	0.093578	0.095595
KEGG_SUL	13	-0.55822	-1.44593	0.077213	0.096052
KEGG_VAL	43	-0.54306	-1.42638	0.129845	0.1054
KEGG_PRO	32	-0.54415	-1.42179	0.120079	0.106973
KEGG_ABC	44	-0.44927	-1.41603	0.082721	0.109401

KEGG_BUT	34	-0.49613	-1.39564	0.121154	0.119934
KEGG_GLY	31	-0.47429	-1.37994	0.104478	0.128483
KEGG_LIM	9	-0.62157	-1.34916	0.165399	0.147388
KEGG_ECM	84	-0.48488	-1.33602	0.206767	0.155227
KEGG_FAT	42	-0.46722	-1.31444	0.190654	0.168707
KEGG_PHE	17	-0.49627	-1.30139	0.149905	0.175659
KEGG_PRI	35	-0.53249	-1.30229	0.24381	0.176003
KEGG_CYT	261	-0.37299	-1.28562	0.192446	0.185931
KEGG_PEN	28	-0.45726	-1.25065	0.202465	0.210964
KEGG_CEL	128	-0.40309	-1.23915	0.251866	0.218558
KEGG_HIS	28	-0.40962	-1.22813	0.211896	0.225555
KEGG_CAR	78	-0.39471	-1.20726	0.271881	0.240309
KEGG_CAL	177	-0.34857	-1.20477	0.244444	0.240663
KEGG_ARR	74	-0.39432	-1.19256	0.266541	0.249073

NOM p-val < 0.05 and FDR q-val < 0.25 were considered as significant
Abbreviations: GSEA, gene set enrichment analysis; CCT3, Chaperonin-containing TCP-1 3
